# Dose and engagement during an extended contact physical activity and dietary behavior change intervention delivered via tailored text messaging: exploring relationships with behavioral outcomes

**DOI:** 10.1186/s12966-021-01179-8

**Published:** 2021-09-07

**Authors:** Brianna S Fjeldsoe, Ana D Goode, Jennifer Job, Elizabeth G Eakin, Kate L Spilsbury, Elisabeth Winkler

**Affiliations:** 1 School of Public Health, Faculty of Medicine, The University of Queensland, Level 4, Herston Road, Herston, Queensland Brisbane, Australia; 2 Centre for Health System Reform and Integration, Mater Research Institute, University of Queensland, Brisbane, Australia; 3 Queensland Academy of Sport, Nathan, Australia

**Keywords:** Text messages, mHealth, Dose, Engagement, Physical activity, Exercise, Diet, Behavior change, Maintenance

## Abstract

**Background:**

Extended contact interventions delivered via text messaging are a low-cost option for promoting the long-term continuation of behavior change. This secondary analysis of a text message–delivered extended contact intervention (‘Get Healthy, Stay Healthy’ (GHSH)) explores the extent to which changes in physical activity, dietary behaviors and body weight were associated with the frequency of text messages (dose) and contact between the health coach and participant (engagement).

**Methods:**

Following a telephone coaching program, participants were randomised to receive extended contact via tailored text messages (GHSH, *n* = 114) or no additional contact (*n* = 114) over a 6-month period. Message dose, timing, and content were based on participant preferences, ascertained during two tailoring telephone calls. All incoming and outgoing messages were recorded. At baseline and 6 months, participants self-reported body weight and dietary behaviors (fruit and vegetable servings/day). Moderate-vigorous physical activity (MVPA) was assessed via accelerometry.

**Results:**

Median dose (25th, 75th percentile) was 53 (33, 72) text messages in total across six months. Mean fortnightly dose in weeks 1–2 was 5.5 (95 % CI: 4.3, 6.6) text messages, and remained stable (with the exception of planned decreases in weeks involving additional intervention contacts). Offset against the average fortnightly dose of goal checks (1.6, 95 % CI: 1.3, 2.0 and 1.5, 95 % CI: 1.2, 1.8, for physical activity and diet respectively), mean replies to goal checks were highest in weeks 1–2 (1.4, 95 % CI: 1.4, 1.5 and 1.3, 95 % CI: 1.2, 1.4, respectively) and tended to become lower in most weeks thereafter. Greater weight loss was positively associated with text message dose (*P* = 0.022), with a difference of 1.9 kg between participants receiving the most and fewest texts. There was no association between engagement and changes in outcome measures.

**Conclusions:**

A fixed dose of texts does not seem suitable to meet participants’ individual preferences. Higher self-selected text doses predicted better weight outcomes. However, greater participant engagement through text replies does not predict more favourable outcomes, despite being a suggested facilitator of successful behavior change maintenance.

**Trial registration:**

Australian New Zealand Clinical Trials Registry number: ACTRN12613000949785. Date registered: 27 August 2013. Retrospectively registered. http://www.anzctr.org.au/.

**Supplementary Information:**

The online version contains supplementary material available at 10.1186/s12966-021-01179-8.

## Background

Behavioral lifestyle interventions can elicit initial improvements in body weight, physical activity and diet in overweight and obese individuals [1, 2]. However, maintaining these improvements in the long-term can be more challenging [3–6]. Despite losing approximately 7–10 % of body weight during initial interventions, individuals typically regain up to half of the weight lost within one year [7]. Increasingly, the need for a long-term solution to improve and maintain lifestyle behaviors is acknowledged [8].

Extension of contact after an initial intervention is a viable and effective method to support the maintenance of physical activity and dietary behavior change [9, 10] and has been shown to reduce the amount of weight regain [1, 11, 12]. However, it is not always feasible to extend interventions involving face-to-face or telephone interactions, due to the substantial cost and time commitment required for both the individual and health coach. Text messaging is an alternative extended contact method, which is low-cost [13], and broad reaching. Tailored information can be efficiently delivered, and two-way communication between individuals and health coaches is possible using minimal resources. Text messaging utilises ‘push’ technology to deliver tailored support in real-time, as individuals go about their daily lives.

A recent meta-analysis on the effectiveness of text message-delivered extended contact interventions on weight management synthesised the limited research in this area [14]. A significant reduction in body weight (-0.82 kg), was evident after extended contact, compared to control conditions [13–16]. Previous meta-analyses of extended contact interventions delivered primarily face-to-face or via telephone, have reported larger pooled effects on body weight of -1.56 kg [11] to -3.2 kg [12], but these modalities are less scalable and more costly than text messaging. When compared to the effects of web-based extended contact interventions on weight loss maintenance in meta-analyses (non-significant reduction in body weight of -0.27 kg) [17], the magnitude of the intervention effect using text messaging supports this communication medium as a more favourable broad-reach, extended contact method.

An understanding of the characteristics of successful health behavior text message-delivered extended contact interventions can help to optimise their design and guide evidence-based practice [18]. It has been reported that effective extended contact interventions deliver tailored text messages; utilise more than four behavior change strategies; and are of at least 12 weeks in duration [14]. Dose and engagement are also particularly important considerations, since text messaging interventions can modify the frequency of text delivery (i.e. dose) and deliver texts that invite a response from individuals (i.e. engagement). In previous studies, dose of text messages varied from once per day (7 text messages per week) [19–21] to less than once per day (≈ 1 to 4 text messages per week) [13, 16, 22, 23]. These previous studies also showed that engagement was inconsistent, with response rates ranging from 20 to 93 % [16, 19–22]. Despite considerable variability in dose and engagement, their relationship with outcome measures has received minimal consideration. Only one study, which individually tailored text message dose based on participant preferences, found that additional text messages were associated with increased physical activity at follow-up [22]. But the same study found there were no associations between text message dose and change in body weight or energy intake [22]. None of the extended contact studies that encouraged two-way text communication investigated whether the response rate (i.e., greater engagement) was associated with more favourable outcomes. However, when text messaging has been utilised to deliver an initial weight loss intervention daily engagement was associated with greater weight loss [24] and it was typically enhanced when there was at least one daily interaction that was initiated by the interventionist [25].

Building on the limited available evidence, this study explores dose and engagement within a tailored, text message-delivered, extended contact intervention targeting body weight, physical activity and dietary behavior. We describe dose and engagement overall and over time, and report on how overall dose and engagement were associated with changes in body weight, accelerometer-measured moderate to vigorous physical activity (MVPA) and a dietary outcome (daily servings of fruits and vegetables).

## Methods

### Overview

The ‘Get Healthy, Stay Healthy’ *(GHSH)* study evaluated a text message-delivered extended care intervention that was offered to eligible participants who completed the ‘Get Healthy Information and Coaching Service’ (GHS) telephone-coaching program. A detailed description of the methods and primary outcomes (including CONSORT checklist) are published elsewhere [15, 26], in brief, the GHSH extended care intervention led to significantly better anthropometric and physical activity outcomes than standard practice (no contact) [15, 26].This secondary analysis reports on data from intervention participants at baseline (upon completion of GHS) and at 6 months (upon completion of GHSH). Recruitment for GHSH began in August 2012 and 6-month follow-up data were collected until March 2014.

### Participants

Participation in GHSH was offered during the final GHS coaching call to all eligible clients who completed the GHS within the recruitment timeframe. Eligibility criteria were: resides in New South Wales, Australia; not intending to re-enroll in GHS coaching; not involved in other GHS evaluations; and, owns a mobile telephone. Participants were randomized 1:1, across two strata (GHS weight loss ≥ 3 kg/<3 kg), to receive the GHSH intervention or control condition. A research assistant with no involvement in participant recruitment conducted the randomization using a randomization website (http://www.randomization.com).

### The Get Healthy, Stay Healthy intervention

The GHSH intervention was delivered via text messaging over a period of approximately 24 weeks. Text messages were tailored based on data that were collected during an initial tailoring telephone call (at baseline) and an interim tailoring call (at approximately week 12) which was used to update preferences. The scripted tailoring calls were conducted by a trained health coach, who asked participants to select a weight goal (maintenance or further loss) and two action areas for behavior change (diet and/or physical activity). For each of the two action areas, the coach asked participants to formulate a specific behavioral goal. For each behavioral goal, participants were asked to identify: rewards for goal attainment; expected benefits; preparatory behaviors; barriers and solutions and, a support person. Participants selected their desired type of texts (from the four types described below), number of text messages of each type (where applicable), and timing of texts (e.g., 6:00 am). The minimum dose a participant could select was three texts per fortnight and the maximum was 13 per fortnight.

### Get Healthy, Stay Healthy text messages

Text messages were generated and sent by research staff, using a purpose-built software package (Propelo™, The University of Queensland, Australia) that enabled the messages to be tailored, pre-programmed and scheduled to be sent at specific times by short messaging service. The text messages, each ≤ 160 characters, were tailored to the participant’s name, gender, goals, identified barriers and strategies, preparatory behaviors, behavioral expectations and nominated support person. As well as sending and receiving texts, the software recorded all incoming and outgoing messages.

Goal reset text messages were sent once in week six and once in week 18 to prompt participants to consider their goals and reset them appropriately. Weight self-monitoring prompts were sent at a fixed frequency of once per fortnight. Participants could elect to receive goal check text messages either once per week or once per fortnight for each behavioral goal (i.e. between 2 and 4 texts per fortnight). These goal checks were interactive, asking participants to reply “yes” or “no” as to whether they achieved their stated goal. Responses were met with a tailored goal check reply text message from the program. Participants were also offered real-time behavioral prompts, which they could elect to receive at a frequency from zero to four per fortnight. These prompts were designed to remind participants of their goals, preparatory behaviors, and anticipated barriers and solutions at times the participant had pre-selected as relevant to receive prompting.

### Control group treatment

To minimize trial attrition, control participants were posted brief written feedback of results following assessments at baseline and 6 months. The control group received no other contact during the intervention period.

### Data collection

The anthropometric and behavioral measurement tools used in this study were the same as those used in the GHS evaluation to enable comparison. More detailed data on dietary behaviors and MVPA were also collected at baseline and 6-months via: a computer-assisted telephone interview (CATI) conducted by a research assistant, and an accelerometer. Sociodemographic data were previously provided during the initial GHS.

#### Anthropometric outcomes

Participants self-reported their body weight in kilograms (while wearing light clothes and no shoes) at baseline and at 6-months. They were encouraged to weigh themselves during the CATI if scales were present; otherwise, they reported their most recent weight. Body Mass Index (BMI) was calculated based on self-reported height and weight at GHS baseline.

#### Dietary behaviors and physical activity

Health behavior data were collected by research staff during the CATI conducted at baseline and at the end of intervention (6 months). Participants were asked to report the number of daily fruit and vegetable servings, using the validated items from the National Nutrition survey [27]. Moderate to vigorous physical activity was assessed via accelerometry (Actigraph GT3X+, Actigraph, Pensacola, FL, USA) using a wear protocol and data reduction procedures described elsewhere [26]. Briefly, participants were asked to wear the monitor on the hip for seven days during waking hours. Based on typical data-reduction procedures, MVPA was measured as the average number of minutes (60-second epochs) per day with ≥ 1952 counts with vertical acceleration [28], excluding invalid days (< 10 h of wear), with non-wear time identified by automated procedures [29].

#### Text message preferences, dose and engagement

Dose and engagement were quantified using the records of incoming and outgoing messages kept by the software package (propelo™), and from the information systematically collected by the health coach during the tailoring calls. Based on these data, dose was measured as the number of text messages received by participants, which depended on both the frequency that participants requested and the duration that participants remained in the 24-week intervention. Dose was quantified separately for each target behavior (diet, physical activity) and overall, and by the purpose of the text (goal checks and real-time behavioral prompts). Engagement was measured in terms of goal check reply rates (replies relative to messages sent). For diet and physical activity, crude reply rates were calculated as number of goal checks to which participants replied divided number of goal checks sent. Overall reply rates (across all goal checks) were calculated as standardised reply rates, by first mean-centring the reply rates for physical activity and diet goal checks, then taking the average. A standardised reply rate of zero would indicate the reply rate is average. This allowed for a more comparable figure between participants who elected to work on both physical activity and diet or only one of those behaviours.

### Data analysis

Analyses were performed in STATA version 14 (StataCorp. Texas, USA) and SPSS version 25 (IBM Corp. Armonl, NY). Text message preferences, dose and engagement were described among all of the randomised participants who set up their text messaging parameters at the initial tailoring interview and went on to receive text messages (n = 111). Fortnightly time trend in dose and engagement were tested using generalized estimating equations (GEE) models. The number of texts received per fortnight were modelled assuming a negative binomial distribution. Reply rates (replies relative to messages sent) were examined using binomial models of goal check replies, offset for the number of goal checks sent. The relationships of dose (number of texts overall, physical activity texts and diet texts) and engagement (standardised reply rate) with behavioral outcomes (changes in diet, physical activity, and weight) were examined using linear regression models, adjusting for randomisation strata and the same set of confounders as the main GHSH evaluation [15]. Following intention-to-treat principles, and due to the relationship between dropout and dose, these models were based on all 114 participants randomised to receive text messaging, with missing data multiply imputed by chained equations (m = 50 imputations). Combined fruit and vegetable intake (which had not been examined as a combined variable in the main evaluation) used the same confounder selection process as the GHSH evaluation and resulted in models being adjusted for (baseline fruit and vegetable intake, randomisation strata, hypertension, smoking and BMI). Not all of the associations were linear and exposures were examined categorically. Accordingly, dose of total texts and engagement were collapsed in tertiles and examined. Dose of physical activity and diet texts were examined as none, low or high, with the latter two categories delineated by a median split of values greater than zero.

## Results

### Participant characteristics

At GHSH baseline, intervention participants (*n* = 114, 65 % female) had a mean age (± SD) of 55.5 (± 12.3) years, a self-report BMI of 29.3 (± 5.8) kg/m^2^, performed 197 (± 144) minutes per week of MVPA, and consumed 3.1 (± 1.4) servings of vegetables and 2.0 (± 0.9) servings of fruit per day, respectively. Further sociodemographic characteristics of the participants have been reported previously [15].

### Text message preferences

Participant text message preferences are shown in Table 1. Three participants withdrew prior to completing the initial tailoring interview (3 %), and therefore set no goals and received no texts; these participants were excluded. The 22 participants who did not complete the 12-week tailoring interview continued to receive texts based on their initial preferences until they either withdrew or completed the program.

**Table. 1:** Participant text message preferences at the initial tailoring interview and the 12 week tailoring interview

		Initial tailoring interview (*n* = 111)			12 week tailoring interview (*n* = 89)		
		All behaviors	Physical activity	Diet	All behaviors	Physical activity	Diet
**Behaviors targeted in goals**	Missing^a^	0	0	0	0	0	0
	Yes	111 (100 %)	100 (90 %)	89 (80 %)	89 (100 %)	79 (89 %)	66 (74 %)
	No	0 (0 %)	11 (10 %)	22 (20 %)	0 (0 %)	10 (11 %)	23 (26 %)
**Goal check texts requested per fortnight**	Missing^a^/NA^b^	0	11	22	0	10	23
	1	-	38 (38 %)	41 (46 %)	-	30 (38 %)	31 (47 %)
	2	46 (41 %)	52 (52 %)	42 (47 %)	41 (46 %)	39 (49 %)	30 (46 %)
	3	25 (23 %)	3 (3 %)	1 (1 %)	18 (20 %)	3 (4 %)	0 (0 %)
	4	40 (36 %)	7 (7 %)	5 (6 %)	30 (33 %)	7 (9 %)	4 (8 %)
**Behavioral prompt texts requested per fortnight**	Missing^a^/NA^b^	0	11	22	0	10	23
	0	58 (52 %)	61 (61 %)	58 (65 %)	45 (50 %)	44 (56 %)	42 (67 %)
	1–2	25 (23 %)	26 (26 %)	24 (27 %)	19 (21 %)	22 (28 %)	19 (29 %)
	3–4	21 (19 %)	13 (13 %)	6 (7 %)	18 (20 %)	11 (14 %)	4 (6 %)
	5–6	4 (4 %)	0 (0 %)	0 (0 %)	5 (6 %)	2 (3 %)	1 (2 %)
	7–8	3 (3 %)	0 (0 %)	1 (1 %)	2 (2 %)	0 (0 %)	0 (0 %)

Footnote: Data are presented as number of participants (% of valid responses), excluding missing/not applicable. Missing^a^: further missing data out of the 111 initial or 90 12 week tailoring interviewees. NA^b^: not applicable as participant did not select to target the behavior in question.

At the first tailoring interview, most participants (*n* = 78, 70 %) chose to work on both physical activity and diet action areas, with less focusing on only physical activity (*n* = 22, 20 %) or only diet (*n* = 11, 10 %). When given the opportunity to change their preferences at the retailoring interview, most (*n* = 82, 92 %), updated the content of their goal in some manner, but most (93 %, *n* = 83) did not alter the focus of their action areas. A number of participants (*n* = 36, 40 %) requested changes to their text messaging schedules. Content changes were requested by some participants via text at the goal resets before (n = 39) and after the retailoring call (*n* = 22).

### Text message dose

Participants (n = 111) received a total text message dose of between 7 and 151 text messages (median [25th, 75th ] percentile: 53 [33, 72]), which included between 4 and 42 goal check text messages (29 [21,41]), and either no behavioral prompts (n = 52, 47 %) or between 6 and 97 prompt texts (24 [18,46]). The total dose of physical text messages received was between 0 and 102 (21 [9,37]), with 0 to 42 goal checks (16 [9,21]) and either no behavioural prompts (n = 47, 42 %) or 5 to 60 (23 [12,36]) behavioural prompts. They received a slightly lower total dose of diet text messages — between 0 and 72 (6 [12,30]) — with 0 to 42 goal checks (12 [4,21]) and either no prompts (n = 36, 32 %) or 4 to 49 (18 [12,24]) behavioural prompts.

**Fig. 1:**
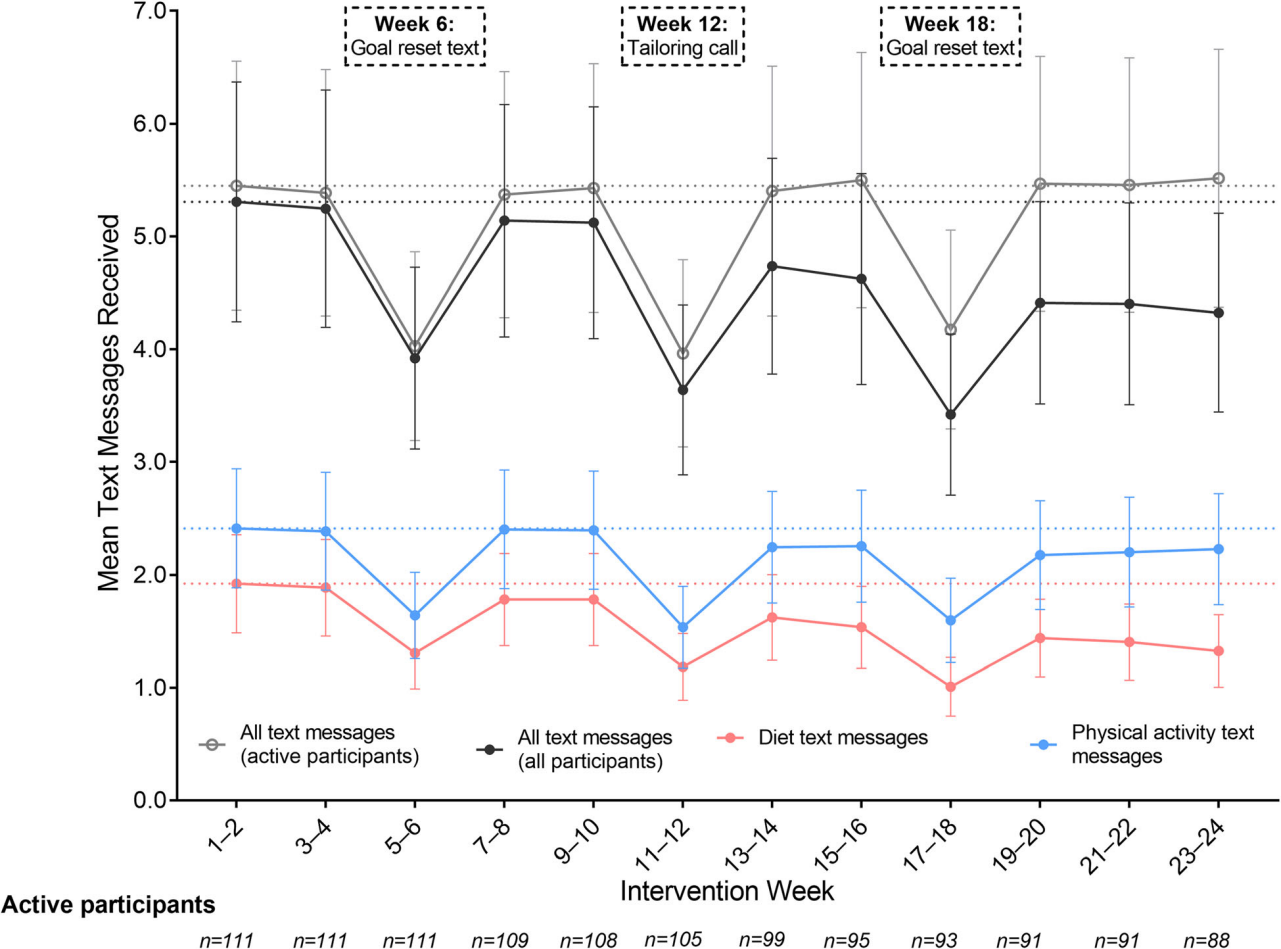
Fortnightly text messaging dose over the 24-week intervention

Fortnightly text messaging dose over time is shown in Fig. 1, overall and for diet and physical activity text messages separately, with numeric data presented in Supplementary Tables 1 and 2. The number of fortnightly texts per participant declined over time, driven by participants withdrawing (and receiving no texts thereafter). Dose per participant still active in the study (n = 111 in weeks 1–2 to n = 88 in weeks 23–24) remained constant over time, apart for drops at weeks 6, 12 and 18, corresponding to the weeks of the goal reset text messages or the 12-week re-tailoring interview. There were more physical activity than diet texts consistently over the 24-week intervention. Mean fortnightly dose was 2.4 (95 % CI: 1.9, 2.9), physical activity text messages per participant initially, and tended to remain at approximately this level throughout the intervention (Fig. 1). Mean fortnightly diet text message receipt declined from 1.9 (95 % CI: 1.5, 2.4) texts per participant initially to a mean of 1.3 (95 % CI: 1.0, 1.7) texts in the final fortnight (Fig. 1), corresponding to participant withdrawal.

### Engagement

**Fig. 2:**
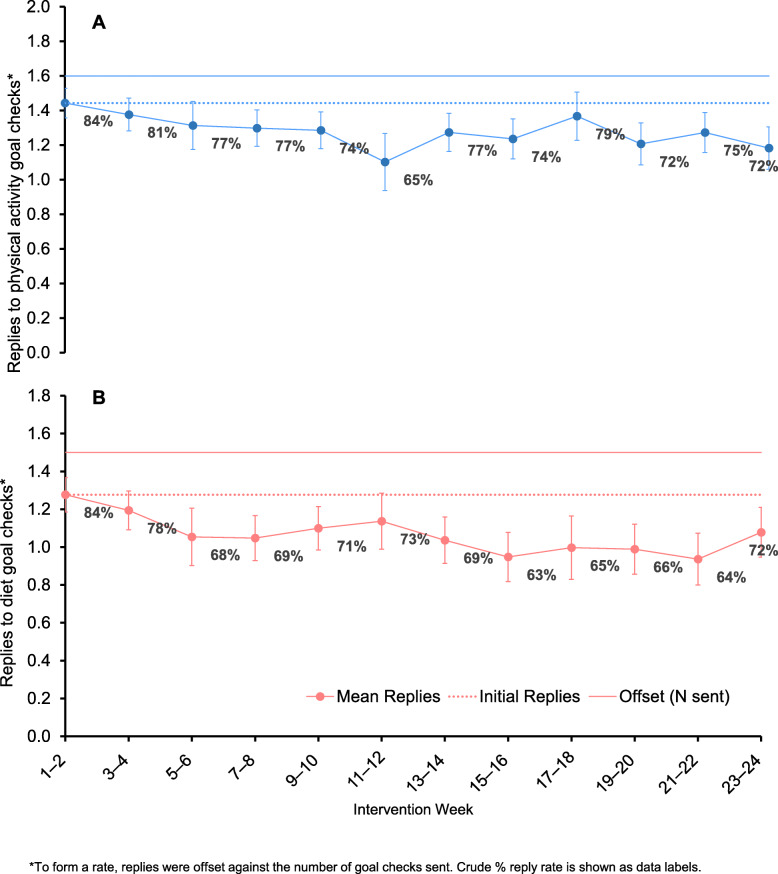
Mean replies to physical activity (A) and diet (B) goal check text messages over time

Engagement is depicted in Fig. 2, with the detailed numeric data presented in the Supplementary Table 3. Offset against the mean number of fortnightly physical activity goal checks (grand mean = 1.6, 95 % CI: 1.3, 2.0), on average there were 1.3 responses (95 % CI: 1.2, 1.4), with replies being highest in weeks 1–2 (1.4, 95 % CI: 1.4, 1.5; 84 %) and the lowest in weeks 11–12 (1.1, 95 % CI: 0.9, 1.3; 65 %) (Fig. 2 A). Relative to the initial replies in the first two weeks, the mean replies to physical activity goal checks were significantly lower from weeks 7–8 onwards, apart from during weeks 17–18 (when a goal reset text was sent). Accounting for the number of diet goal checks sent (grand mean = 1.5, 95 % CI: 1.2, 1.8), the mean number of replies were also highest in weeks 1–2 (1.3, 95 % CI: 1.2, 1.4; 84 %), with a grand mean of 1.1 (95 % CI: 1.0, 1.2) replies (Fig. 2B). Replies were significantly lower than weeks 1–2 during all of the weeks from weeks 5–6 onwards, apart from weeks 11–12 when the re-tailoring call occurred (Fig. 2B).

### Relationships of dose and engagement with anthropometry and behavior change

Anthropometric and behavior change results are reported in detail elsewhere [15]. Associations of dose and engagement with changes in body weight, physical activity and diet are shown in Table 2. Greater weight loss was positively associated with number of text messages (*P* = 0.022), with a difference of 1.9 kg between those receiving the most and fewest texts (95 % CI: 0.3, 3.4). A positive association between text message dose and weight loss was also observed when considering the number of texts related to physical activity (-1.6 kg, 95 % CI: 0.2, 2.9, *P* = 0.022) and related to diet (-2.1 kg, 95 % CI: 0.6, 3.5, *P* = 0.006) separately. This was observed only when comparing high and low doses, not when comparing a high dose with no dose at all (which may have been due to a participant’s focus on the alternative behavior or to receiving no texts). Those who elected to receive behavioral prompts achieved non-significantly greater weight loss (-1.0 kg, 95 % CI: -2.2, 0.3) than those who did not. There was a tendency for those receiving lower doses (overall texts and physical activity texts) to have smaller increases in MVPA, by approximately 20 to 30 min/week than those receiving the highest doses, but associations were statistically non-significant. Receiving no dietary texts (e.g., only receiving physical activity texts or due to withdrawing) was associated with a greater increase in MVPA of 67 min/week (95 % CI: 1, 133, *P* = 0.046). There was no significant association between text message dose and changes in fruit and vegetable intake; however effects as large as one serving/day were within the 95 % confidence intervals. Engagement did not show any sizeable or statistically significant association with changes in body weight, or servings of fruits and vegetables. Relative to those replying to goal checks most often, participants in the middle and lowest categories of engagement tended to have greatest increases in MVPA (+ 40 min/week and + 45 min/week, respectively), but the difference did not reach statistical significance.

**Table. 2:** Associations of text message dose and engagement with changes in weight, physical activity and diet

Text message Characteristic		Body weight(kg)		MVPA(min/week)		Fruit and vegetables (servings/day)	
	n	Beta(95 % CI)	*p*-value	Beta(95 % CI)	*p*-value	Beta(95 % CI)	*p*-value
**Dose**							
** Number of texts (all)**							
High (top 33 %), ≥ 67	38	ref		ref		ref	
Medium, 36–66	38	1.2 (-0.3, 2.7)	0.113	-9 (-62, 44)	0.750	-0.1 (-0.9, 0.6)	0.704
Low (bottom 33 %),<36	38	1.9 (0.3, 3.4)	0.022	-29 (-91, 34)	0.367	0.1 (-0.7, 0.9)	0.801
*p-value for trend*			0.022		0.368		0.786
** Number of texts (physical activity)**							
High (top 50 %), ≥ 24	45	ref		ref		ref	
Low (bottom 50 %), 1–23	57	1.6 (0.2, 2.9)	0.022	-11 (-59, 37)	0.642	-0.3 (-1.0, 0.3)	0.339
None	12	0.2 (-2.3, 2.8)	0.848	-25 (-119, 70)	0.604	-0.4 (-1.6, 0.9)	0.551
*p-value for trend*			0.208		0.534		0.368
** Number of texts (diet)**							
High (top 50 %), ≥ 24	34	ref		ref		ref	
Low (bottom 50 %), 1–23	55	2.1 (0.6, 3.5)	0.006	-7 (-60, 46)	0.795	-0.6 (-1.3, 0.05)	0.067
None	25	0.5 (-1.3, 2.4)	0.584	67 (1.1, 133)	0.046	0.5 (-0.4, 1.4)	0.262
*p-value for trend*			0.430		0.055		0.422
** Behavioral prompt texts**							
Received versus notreceived	59 vs. 55	-1.0 (-2.2, 0.3)	0.131	-2.3 (-53, 48)	0.929	0.1 (-0.5, 0.8)	0.688
**Engagement**							
** Standardised reply rate**							
High (top 33 %), ≥ 14.6 %	36	ref		ref		ref	
Medium, -1.6–14.6 %	38	0.4 (-1.2, 2.0)	0.591	40 (-13, 93)	0.139	-0.0 (-0.8, 0.7)	0.958
Low (bottom 33 %), <-1.6 %	40	-0.1 (-1.7, 1.5)	0.877	45 (-9, 98)	0.101	0.1 (-0.7, 0.8)	0.842
*p-value for trend*			0.868		0.104		0.839

Footnote: Table reports regression coefficient and 95 % confidence interval, as assessed via linear regression models, adjusting for baseline values, randomisation strata and confounders. *P*-value for trend shows p for trend across categories of high medium and low. Abbreviations: *n* number of participants, *MVPA* moderate-vigorous physical activity.

## Discussion

We report in detail on dose and engagement within a tailored text-message delivered extended contact intervention for lifestyle behavior, in which participants could self-select their dosage and were able to engage with the program by replying to texts. The selected text message dose was variable between participants, suggesting that a fixed dose is unsuitable to meet participant preferences in extended contact interventions. However, there appeared to be a limited need to re-tailor dosage over the 24-week intervention, with many participants opting to retain their initial dose preferences. Consequently, dose remained stable over time within the participants still active in the program, but declined overall from a number of participants withdrawing (and thus receiving no text messages). Engagement varied significantly over time, with reply rates to goal check messages tending to be highest initially and lower in subsequent weeks. A higher text message dose was associated with significantly better body weight outcomes, but there was no statistically significant relationship between engagement and weight, diet or physical activity outcome measures.

In this general population of free-living adults, participants who received a higher dose of text messages overall achieved approximately 2 kg greater weight loss than those receiving the lowest dose, with similar findings seen for diet and physical activity texts. By contrast, in a previous study examining a small sample of breast cancer survivors, a non-significant tendency for lesser weight loss with greater self-selected doses of text messages was reported [22]. Greater physical activity changes tended to correspond with receiving no dietary texts (i.e., focusing exclusively on physical activity), with a substantial but non-significant effect seen. This effect may be explained by participants solely focusing on physical activity because they were more motivated to be physically active, or because offering the option to choose a single behavior focus in the intervention offers an alternative for those who find focusing on two behaviors overwhelming [30] No large or significant associations were evident between any of the dose measures and fruit and vegetable intake, however wide confidence intervals indicated meaningful effects may have been missed for this outcome.

When considering the associations between higher dosage and better outcomes it is important to consider that the higher dosages were self-selected by participants. An explanation of this finding is that the better weight outcomes seen for those selecting a higher dose may have been confounded by the fact that these participants were more motivated than those selecting a lower dose. However, the same relationship has been seen in studies comparing higher and lower text message doses [31], lending some credence to the idea that the benefit of higher dosage may be at least in part due to greater frequency of contact itself, not only the type of participants who choose to have the most frequent contact. However, selecting the highest possible dose to improve outcomes may not be the best strategy if these dosages are higher than participants prefer. Whilst repeated exposures to messages may have led to increased effectiveness, too many messages and excessive repetition may become burdensome to participants and reduce acceptability of programs [32]. The current study and other studies using flexible or participant-set doses [22] suggest that many participants would prefer a dose that is lower than one or more messages per day, which is the dose often set in initial lifestyle [33–41] and extended contact [19–21] text messaging interventions. and the multiple messages per day seen in the most effective studies [31].

In GHSH, participants received on average, self-selected doses of 4 to 6 texts per fortnight (2 to 3 per week), which was at the lower end of the range of doses offered (i.e. 3 to 13 texts per fortnight). Participants in previous lifestyle interventions have averaged self-selected doses slightly higher than GHSH participants, including: 3.7 [42], 4.0 [22] 4.4 [43] and 5.1 [44] texts per week. The strict maximum limit of two goal checks and one weight self-monitoring prompt per fortnight may have contributed to the lower self-selected dose in GHSH compared to previous literature. Despite the opportunity to reset the dose, total dose remained stable over time while participants were actively in the program, suggesting that the possible benefits of retailoring dosage or tapering dosage over time may have little relevance over a period as short as 24 weeks. Taken together, these findings suggest that the text message dose in extended contact interventions may influence weight loss outcomes, but in order to satisfy participant preferences, it is necessary to enable participants to self-select dose. Future interventions should aim to identify an evidence-based minimum and maximum dose range for participants to select from.

Previous text message-delivered interventions targeting initial weight loss have seen associations between engagement and outcomes [37, 41]. In our extended contact intervention, we did not observe any statistically significant relationship between engagement and changes in physical activity, diet or body weight. One factor may have been insufficient sample size, as confidence intervals contained some potentially sizeable differences. Also, goal check reply rates are limited as an indicator of engagement; they may be influenced by many considerations apart from participant’s level of interest in the program, such as communication style and behavioural expectations.

In the literature, overall degree of engagement has been variable, with message response rates ranging from 20 to 93 % [16, 19–22]. In the present study, fortnightly reply rates were fairly high through the 24 week period (63–84 %), but did decline significantly over time. Other lifestyle text-messaging interventions have also seen declining reply rates over time [36, 39, 45, 46]. This could be a general process of participants disengaging with text messages or could relate to some specific features of the messaging. The GHSH intervention protocol used a semi-automated response system, which tailored the goal check reply based on the yes/no component of the participants text and did not acknowledge additional information which may have been offered by the participants (e.g., elaborating on how they were feeling or a specific achievement during the week). An absence of the health coach acknowledging their personal experiences (as a coach would have done during a telephone call) may have led participants to disengage. Indeed, post-intervention qualitative interviews with 62 participants found that whilst most liked the GHSH program, the most common reason for not like the texting program was that it felt too automated [15]. This study did not see a significant association between participant reply rates and behavioral outcomes, however this is not to say that an absence of two-way communication would be unimportant to participant outcomes. Two-way communication should remain an important component of text-message delivered extended contact interventions as other literature supports that it maintains participants’ perceptions of accountability and a personal connection with their health coach [15], both of which are common features of successful extended contact interventions for weight loss maintenance [12]. This finding touches on an important tension in mHealth programs between personally tailoring two-way communications to maintain accountability and rapport whilst still designing scalable programs requiring minimal ongoing human management [47]. As mHealth behaviour change interventions embrace technological advances, such as.

platforms enabling automated rule-based tailoring and artificial intelligence, we may be able to more readily meet both needs [48].

## Strengths and limitations

A strength of the study was the wide range of choices participants were offered initially and then re-offered at the interim tailoring call. This provided novel information regarding both participant preferences throughout a text-messaging intervention, and a detailed examination of the relationship between these preferences and the anthropometric and behavioral outcomes attained. A limitation was that the study was not powered *a priori* on this question. It appeared underpowered, since several statistically non-significant associations were potentially of a meaningful magnitude (≥ 2 kg body weight, ≥ 60 min/week MVPA or ≥ 1 serving of fruit or vegetables per day), based on the confidence intervals.

## Conclusions

In the context of a tailored, text-message delivered, extended contact intervention that targeted weight loss, diet and physical activity, higher self-selected text message doses predicted better weight outcomes. This benefit was achieved regardless of whether the messages targeted diet or physical activity (as per the participant’s selection). This study did not confirm other literature supporting a relevance of engagement with an intervention for ultimate behavioral success. Two key potential reasons for this may be the different context (extended care versus initial intervention) and the engagement indicator (text messaging reply rates). There was a large variation in the doses participants self-selected. This would suggest that a fixed dose is unsuitable for meeting participant preferences.

## Supplementary Information

**Additional file 1:**

**Additional file 2:**

**Additional file 3:**

## Data Availability

The datasets analysed during the current study are available from the corresponding author on reasonable request.

## Abbreviations

BMI: Body mass index
CATI: Computer-assisted telephone interview
CI: Confidence interval
GEE: Generalized estimating equations
GHS: Get Healthy Service
GHSH: Get Healthy, Stay Healthy intervention
MVPA: Moderate-vigorous physical activity
SD: Standard deviation
