# Supplementary material for: Dose and engagement during an extended contact physical activity and dietary behavior change intervention delivered via tailored text messaging: exploring relationships with behavioral outcomes
Source: Int J Behav Nutr Phys Act. 2021 Sep 7;18:119. doi: 10.1186/s12966-021-01179-8 (PMC8425069; doi:10.1186/s12966-021-01179-8)
Supplement: Supplementary file 1 — Additional file 1: [file 12966_2021_1179_MOESM1_ESM.docx]

Table: Fortnightly text messages sent (active participants)

| **Study Week** |  | **All** | | | **Physical Activity** | | | **Diet** | | |
| --- | --- | --- | --- | --- | --- | --- | --- | --- | --- | --- |
|  | **n** | **Mean (95% CI)** | **Difference (95% CI)** | **p** | **Mean (95% CI)** | **Difference (95% CI)** | **p** | **Mean (95% CI)** | **Difference (95% CI)** | **p** |
| 1–2 | 111 | 5.45 (4.35, 6.55) |  |  | 2.48 (1.93, 3.02) | 0 (ref) |  | 1.97 (1.52, 2.42) | 0 (ref) |  |
| 3–4 | 111 | 5.39 (4.30, 6.48) | 0.00 (-0.02, 0.02) | 0.844 | 2.45 (1.91, 2.99) | 0.00 (-0.03, 0.03) | 0.830 | 1.94 (1.49, 2.38) | -0.01 (-0.05, 0.04) | 0.787 |
| 5–6 | 111 | 4.03 (3.19, 4.86) | **-0.05 (-0.08, -0.03)** | **<0.001** | 1.68 (1.29, 2.08) | **-0.13 (-0.17, -0.09)** | **<0.001** | 1.34 (1.01, 1.67) | **-0.15 (-0.21, -0.09)** | **<0.001** |
| 7–8 | 109 | 5.37 (4.28, 6.46) | 0.00 (-0.02, 0.02) | 0.807 | 2.51 (1.96, 3.06) | 0.00 (-0.03, 0.03) | 0.808 | 1.86 (1.43, 2.29) | -0.02 (-0.07, 0.03) | 0.402 |
| 9–10 | 108 | 5.43 (4.33, 6.53) | 0.00 (-0.02, 0.02) | 0.949 | 2.54 (1.98, 3.10) | 0.01 (-0.02, 0.04) | 0.622 | 1.89 (1.45, 2.32) | -0.01 (-0.06, 0.03) | 0.527 |
| 11–12 | 105 | 3.96 (3.13, 4.79) | **-0.06 (-0.08, -0.03)** | **<0.001** | 1.67 (1.28, 2.07) | **-0.13 (-0.17, -0.09)** | **<0.001** | 1.29 (0.97, 1.61) | **-0.16 (-0.23, -0.10)** | **<0.001** |
| 13–14 | 99 | 5.40 (4.30, 6.51) | 0.00 (-0.02, 0.02) | 0.886 | 2.54 (1.98, 3.11) | 0.01 (-0.02, 0.04) | 0.622 | 1.86 (1.42, 2.29) | -0.02 (-0.07, 0.03) | 0.397 |
| 15–16 | 95 | 5.50 (4.37, 6.63) | 0.00 (-0.02, 0.02) | 0.883 | 2.67 (2.08, 3.25) | 0.02 (-0.01, 0.05) | 0.172 | 1.83 (1.40, 2.26) | -0.03 (-0.07, 0.02) | 0.307 |
| 17–18 | 93 | 4.17 (3.29, 5.06) | **-0.05 (-0.07, -0.02)** | **<0.001** | 1.89 (1.45, 2.33) | **-0.09 (-0.12, -0.05)** | **<0.001** | 1.28 (0.96, 1.61) | **-0.17 (-0.23, -0.10)** | **<0.001** |
| 19–20 | 91 | 5.47 (4.34, 6.60) | 0.00 (-0.02, 0.02) | 0.961 | 2.61 (2.03, 3.19) | 0.01 (-0.02, 0.04) | 0.335 | 1.85 (1.41, 2.29) | -0.02 (-0.07, 0.03) | 0.379 |
| 21–22 | 91 | 5.46 (4.33, 6.58) | 0.00 (-0.02, 0.02) | 0.986 | 2.64 (2.06, 3.23) | 0.02 (-0.01, 0.05) | 0.236 | 1.81 (1.38, 2.23) | -0.03 (-0.08, 0.02) | 0.227 |
| 23–24 | 88 | 5.52 (4.37, 6.66) | 0.00 (-0.02, 0.02) | 0.850 | 2.69 (2.09, 3.28) | 0.02 (-0.01, 0.05) | 0.138 | 1.81 (1.38, 2.24) | -0.03 (-0.08, 0.02) | 0.256 |
| Overall ^a^ |  | 5.09 (4.13, 6.04) |  | **<0.001** | 2.35 (1.86, 2.85) |  | **<0.001** | 1.73 (1.36, 2.10) |  | **<0.001** |

Table presents means (95% CI) and difference from generalised estimating equations models (negative binomial distribution)

^a^ Grand mean (95%CI) or overall p value.
