# Supplementary material for: Dose and engagement during an extended contact physical activity and dietary behavior change intervention delivered via tailored text messaging: exploring relationships with behavioral outcomes
Source: Int J Behav Nutr Phys Act. 2021 Sep 7;18:119. doi: 10.1186/s12966-021-01179-8 (PMC8425069; doi:10.1186/s12966-021-01179-8)
Supplement: Supplementary file 2 — Additional file 2: [file 12966_2021_1179_MOESM2_ESM.docx]

Table: Time trends in the number of fortnightly text messages sent to text message recipients (n=111)

| **Study Week** | **All** | | | **Physical Activity** | | | **Diet** | | |
| --- | --- | --- | --- | --- | --- | --- | --- | --- | --- |
|  | **Mean (95% CI)** | **Difference (95% CI)** | **p** | **Mean (95% CI)** | **Difference (95% CI)** | **p** | **Mean (95% CI)** | **Difference (95% CI)** | **p** |
| 1–2 | 5.31 (4.24, 6.37) | 0 (ref) |  | 2.41 (1.89, 2.94) | 0 (ref) |  | 1.92 (1.49, 2.36) | 0 (ref) |  |
| 3–4 | 5.25 (4.19, 6.30) | 0.00 (-0.03, 0.02) | 0.877 | 2.39 (1.86, 2.91) | 0.00 (-0.04, 0.03) | 0.863 | 1.89 (1.46, 2.31) | -0.01 (-0.06, 0.05) | 0.823 |
| 5–6 | 3.92 (3.11, 4.73) | **-0.05 (-0.08, -0.03)** | **<0.001** | 1.64 (1.26, 2.02) | **-0.13 (-0.18, -0.08)** | **<0.001** | 1.31 (0.99, 1.63) | **-0.15 (-0.22, -0.08)** | **<0.001** |
| 7–8 | 5.14 (4.11, 6.17) | -0.01 (-0.03, 0.02) | 0.671 | 2.40 (1.88, 2.93) | 0.00 (-0.04, 0.04) | 0.954 | 1.78 (1.37, 2.19) | -0.03 (-0.08, 0.03) | 0.361 |
| 9–10 | 5.12 (4.09, 6.15) | -0.01 (-0.03, 0.02) | 0.639 | 2.39 (1.87, 2.92) | 0.00 (-0.04, 0.03) | 0.909 | 1.78 (1.37, 2.19) | -0.03 (-0.08, 0.03) | 0.361 |
| 11–12 | 3.64 (2.89, 4.39) | **-0.07 (-0.10, -0.04)** | **<0.001** | 1.54 (1.17, 1.90) | **-0.15 (-0.21, -0.10)** | **<0.001** | 1.18 (0.89, 1.48) | **-0.19 (-0.27, -0.12)** | **<0.001** |
| 13–14 | 4.74 (3.78, 5.69) | -0.02 (-0.04, 0.01) | 0.135 | 2.25 (1.75, 2.74) | -0.02 (-0.06, 0.02) | 0.266 | 1.62 (1.24, 2.00) | **-0.06 (-0.12, 0.00)** | **0.047** |
| 15–16 | 4.62 (3.69, 5.56) | -0.02 (-0.05, 0.00) | 0.071 | 2.25 (1.76, 2.75) | -0.02 (-0.06, 0.02) | 0.292 | 1.54 (1.17, 1.90) | **-0.08 (-0.14, -0.02)** | **0.009** |
| 17–18 | 3.42 (2.71, 4.13) | **-0.08 (-0.12, -0.05)** | **<0.001** | 1.60 (1.22, 1.97) | **-0.14 (-0.19, -0.09)** | **<0.001** | 1.01 (0.75, 1.27) | **-0.27 (-0.36, -0.18)** | **<0.001** |
| 19–20 | 4.41 (3.52, 5.31) | **-0.03 (-0.06, -0.01)** | **0.017** | 2.18 (1.69, 2.66) | -0.03 (-0.07, 0.01) | 0.111 | 1.44 (1.09, 1.78) | **-0.11 (-0.17, -0.04)** | **0.001** |
| 21–22 | 4.40 (3.51, 5.30) | **-0.03 (-0.06, -0.01)** | **0.016** | 2.20 (1.71, 2.69) | -0.03 (-0.07, 0.01) | 0.158 | 1.40 (1.07, 1.74) | **-0.12 (-0.19, -0.05)** | **<0.001** |
| 23–24 | 4.32 (3.44, 5.21) | **-0.04 (-0.06, -0.01)** | **0.009** | 2.23 (1.74, 2.72) | -0.02 (-0.06, 0.01) | 0.218 | 1.32 (1.00, 1.65) | **-0.14 (-0.21, -0.07)** | **<0.001** |
| Overall ^a^ | 4.52 (3.73, 5.32) |  | **<0.001** | 2.12 (1.69, 2.56) |  | **<0.001** | 1.52 (1.20, 1.83) |  | **<0.001** |

Table presents means (95% CI) and difference from generalised estimating equations models (negative binomial distribution)

^a^ Grand mean (95%CI) or overall p value.
