# Supplementary material for: Dose and engagement during an extended contact physical activity and dietary behavior change intervention delivered via tailored text messaging: exploring relationships with behavioral outcomes
Source: Int J Behav Nutr Phys Act. 2021 Sep 7;18:119. doi: 10.1186/s12966-021-01179-8 (PMC8425069; doi:10.1186/s12966-021-01179-8)
Supplement: Supplementary file 3 — Additional file 3: [file 12966_2021_1179_MOESM3_ESM.docx]

Table: Fortnightly replies to goal check text messages over time, among participants actively receiving goal checks

| **Study Week** | **n** | **Physical Activity** | | | **Diet** | | |
| --- | --- | --- | --- | --- | --- | --- | --- |
|  |  | **Mean (95% CI)** | **Difference (95% CI)** | **p** | **Mean (95% CI)** | **Difference (95% CI)** | **p** |
| 1–2 | 111 | 0.86 (0.81, 0.91) | 0 (ref) |  | 0.83 (0.77, 0.89) | 0 (ref) |  |
| 3–4 | 111 | 0.82 (0.76, 0.88) | -0.30 (-0.77, 0.18) | 0.219 | 0.78 (0.71, 0.85) | -0.35 (-0.80, 0.11) | 0.134 |
| 5–6 | 111 | 0.78 (0.70, 0.87) | -0.54 (-1.08, 0.01) | 0.053 | 0.69 (0.59, 0.79) | **-0.82 (-1.33, -0.32)** | **0.001** |
| 7–8 | 109 | 0.77 (0.71, 0.84) | **-0.59 (-1.05, -0.12)** | **0.013** | 0.68 (0.61, 0.76) | **-0.84 (-1.28, -0.40)** | **<0.001** |
| 9–10 | 108 | 0.77 (0.70, 0.83) | **-0.63 (-1.09, -0.17)** | **0.007** | 0.72 (0.64, 0.79) | **-0.68 (-1.13, -0.23)** | **0.003** |
| 11–12 | 105 | 0.66 (0.56, 0.76) | **-1.17 (-1.68, -0.65)** | **<0.001** | 0.74 (0.65, 0.84) | **-0.55 (-1.09, -0.02)** | **0.043** |
| 13–14 | 99 | 0.76 (0.69, 0.82) | **-0.67 (-1.14, -0.20)** | **0.005** | 0.68 (0.60, 0.76) | **-0.87 (-1.33, -0.42)** | **<0.001** |
| 15–16 | 95 | 0.74 (0.67, 0.81) | **-0.79 (-1.26, -0.32)** | **0.001** | 0.62 (0.53, 0.70) | **-1.13 (-1.58, -0.68)** | **<0.001** |
| 17–18 | 93 | 0.81 (0.73, 0.90) | -0.34 (-0.93, 0.26) | 0.268 | 0.65 (0.54, 0.76) | **-0.99 (-1.52, -0.46)** | **<0.001** |
| 19–20 | 91 | 0.72 (0.65, 0.79) | **-0.88 (-1.35, -0.40)** | **<0.001** | 0.65 (0.56, 0.73) | **-1.01 (-1.47, -0.55)** | **<0.001** |
| 21–22 | 91 | 0.76 (0.69, 0.83) | **-0.67 (-1.15, -0.19)** | **0.006** | 0.61 (0.52, 0.70) | **-1.16 (-1.62, -0.70)** | **<0.001** |
| 23–24 | 88 | 0.70 (0.63, 0.78) | **-0.95 (-1.42, -0.48)** | **<0.001** | 0.70 (0.62, 0.79) | **-0.75 (-1.23, -0.26)** | **0.003** |
| Overall sent ^a^ |  | 1.62 (1.26, 1.97) |  | **<0.001** | 1.49 (1.15, 1.82) |  | <0.001 |

Table presents means (95% CI) and difference from generalised estimating equations models (negative binomial distribution) for number of replies offset for number of goal check texts sent.

^a^ Mean number of goal checks sent - Grand mean (95%CI) or overall p value.
